# Citrus Production Under Screen as a Strategy to Protect Grapefruit Trees From Huanglongbing Disease

**DOI:** 10.3389/fpls.2019.01598

**Published:** 2019-12-18

**Authors:** Rhuanito S. Ferrarezi, Jawwad A. Qureshi, Alan L. Wright, Mark A. Ritenour, Natalia P. F. Macan

**Affiliations:** ^1^ Indian River Research and Education Center, Institute of Food and Agricultural Sciences, University of Florida, Fort Pierce, FL, United States; ^2^ Southwest Florida Research and Education Center, Institute of Food and Agricultural Sciences, University of Florida, Immokalee, FL, United States

**Keywords:** enclosure, potted tree production, high-density, fertigation, vector exclusion

## Abstract

Citrus production under enclosed structures can exclude the Asian citrus psyllid (ACP, *Diaphorina citri*) and eliminate the negative effects of citrus greening or huanglongbing (HLB) disease caused by *Candidatus* Liberibacter asiaticus to the grapefruit (*Citrus paradisi*) fresh fruit industry. Physically impeding the insect vector from accessing trees is a logical method to have disease-free groves. Our objectives were to assess the ability of enclosed screenhouses to exclude the ACP, stop HLB inoculation and dissemination, and improve fruit yield of in-ground and container-grown 6-year-old “Ray Ruby” grapefruit at super-high planting densities relative to open-air trees. We built a large structure to allow commercial-scale trials and tested two production systems (screenhouse and open-air), two planting systems (in-ground and potted), and two rootstocks (“Sour Orange” [*Citrus × aurantium*] and “US-897” [*Citrus reticulata* × *Poncirus trifoliate*]). The experimental design was a randomized complete block design split-split-plot with four replications. Four passively ventilated 1,080-m^2^ completely enclosed screenhouses were constructed using a 50-mesh monofilament high-density polyethylene screen. The main support for each enclosed, covered structure consisted of pressure-treated, wooden utility poles. Trees were planted in Sept/2013 on a density of 1,957 trees/ha. Irrigation was performed on-demand using two 7.6-LPH drip emitters per tree, and fertigation was applied three times/week using 15N-2.6P-22.4K water-soluble fertilizer at 180 kg N/ha. Psyllids were monitored using sticky cards and detected inside the screenhouses post-Hurricane Irma, which damaged the screen structures in Sept/2017, leaving openings until repairs were completed in Apr/2018. Screen aging and a tropical storm in April/2019 caused another major screen opening fixed in Oct/2019. Despite the weather-related damages to the screens, only trees cultivated in open-air tested positive for *Candidatus* Liberibacter asiaticus after 6 years. There was fast disease progression for all outside treatments, with 100% infection. Covered, in-ground trees exhibited the highest trunk diameter and canopy volume (*P* < 0.0001). Trees grown inside screenhouses exhibited higher fruit yield than outside trees, with the highest yield observed for in-ground trees on “US-897” (51,081 kg/ha) (*P* < 0.0001). Several open-air treatments particularly in containers did not produce any fruit. On the other hand, potted grapefruit trees cultivated inside the enclosures had the highest soluble solids content (*P* < 0.001). The screenhouses provided disease exclusion, increased fruit yield, and fruit quality, representing an alternative for growers interested in producing high-quality fruit for the fresh market. Production cost and economic viability still need to be evaluated for large-scale implementation.

## Introduction

Citrus is directly linked with the “Sunshine State” agricultural identity. Unfortunately, Florida’s total citrus production declined 83% from 291.8 million boxes in 2003/04 to 49.58 million boxes in 2017/18, indicating a severe crisis in the fruit market (U.S Department of Agriculture, 2019). Citrus bearing acreage also declined drastically from 679,000 acres in 2003/04 to 400,900 acres in 2017/18. On-tree citrus values dropped 47% from $1.046B in 2008/09 to $0.551B in 2017/18 (U.S Department of Agriculture, 2019). Florida grapefruit production for the fresh market experienced an even steeper decline, dropping by 90% from 1.738 million tons in 2003/04 to 0.165 million tons in 2017/18. Grapefruit (*Citrus paradisi*) bearing acreage also reduced from 82,300 acres in 2003/04 to 29,800 acres in 2017/18 (U.S Department of Agriculture, 2019).

This devastating decline was first caused by State efforts to eradicate citrus canker (*Xanthomonas axonopodis* pv. citri), followed by catastrophic hurricanes in 2004 and 2005 (Ferrarezi et al., 2019), and then by the devastating citrus greening disease or huanglongbing (HLB), associated with the phloem-plugging bacterium *Candidatus* Liberibacter asiaticus (*C*Las). Eradication efforts for citrus canker were halted in 2006, while HLB has spread throughout Florida, with more than 80% of the citrus trees currently infected by the disease.

HLB is transmitted by the Asian citrus psyllid (ACP, *Diaphorina citri*) (Bové, 2006). The vector is a small 3 to 4 mm long insect. Shoots with newly developing tender leaves are needed by the females for oviposition and nymphal development; whereas adults can survive but not reproduce over winter, on mature leaves (Qureshi and Stansly, 2009). The adults require feeding on young shoots to develop and reproduce. Insect development is temperature-dependent. Under optimal conditions psyllids can go through 10–12 generations each year in Florida (Qureshi and Stansly, 2009; Qureshi and Stansly, 2010). Nymphs go through five nymphal instars to develop to adulthood in approximately 2 weeks. *Diaphorina citri* has a high reproductive rate and a single female is capable of laying several hundred eggs. The intrinsic rate of population increase in the absence of any biotic mortality factors was estimated in the range of 125–285 (Qureshi and Stansly, 2009). Nymphs and adults cause direct damage to the plants by feeding on the young shoots and leaves. Both biological and chemical methods of control provide significant reduction in the psyllid populations (Qureshi et al., 2009; Qureshi and Stansly, 2009; Qureshi and Stansly, 2010; Qureshi et al., 2014).

The planting of HLB-free trees from commercial nurseries, early removal of infected trees, application of foliar bactericides, and vector control are the most used strategies to manage the disease in the field (Dala-Paula et al., 2019). Scouting and frequent spraying of insecticides constitute the core of mitigation programs to reduce ACP population, reducing the feeding activity and the disease transmission (Stansly et al., 2014). In Brazil, frequent and coordinated insecticide application in area-wide programs is a successful strategy to manage the ACP (Bassanezi et al., 2013). Geographical isolation and mountainous landscape also naturally reduce the ACP population. In the United States, specifically in Florida, those strategies were tested through the Citrus Health Management Areas (CHMAs) and were not successful due to the environmental conditions and extremely high psyllid population (Singerman et al., 2017).

Despite the use of aggressive vector management programs, it has been virtually impossible to reduce the spread of the HLB in Florida. Completely enclosed screenhouses can physically exclude the vector and prevent disease transmission (Ferrarezi et al., 2017b). The screen acts as a physical barrier, blocking psyllids and insects larger than 50 mesh (0.297 mm) from damaging citrus trees (Ferrarezi et al., 2017a). The system provides a shaded environment with reduced solar radiation and evapotranspiration, creating an ideal environment for vigorous plant growth, increased fruit yield and improved fruit quality (Ferrarezi et al., 2017a). An HLB-free environment allows the production of high-quality fresh fruit, which retails for a high cash price due to the current high demand and shortage in supply.

The system originated at the University of Florida’s Institute of Food and Agricultural Sciences (UF/IFAS) Indian River Research and Education Center in Fort Pierce, FL and has successfully been tested at the UF/IFAS Citrus Research and Education Center in Lake Alfred, FL as well (Ferrarezi et al., 2017b). The economics of citrus under protective screens (CUPS) is being determined (Schumann and Singerman, 2016). To date, there are approximately 161 ha of commercial CUPS in Florida, and 60 ha are planned (Eduardo Pines and Steven Callaham, personal communication).

The disadvantages of growing citrus indoors are related to the increase the populations of certain pests such as citrus rust mites (*Phyllocoptruta oleivora*), snow scales (*Unaspis citri*), and thrips (*Frankliniella* spp.), and diseases like citrus greasy spot (*Mycosphaerella citri*). Screenhouse construction costs are still high at approximately $10.76 per square meter, which does not include irrigation and trees. The 40- to 50-mesh high-density polyethylene screen may need replacement every 5 years (up to $5.38 per square meter) (Schumann et al., 2017).

Previous studies reported on the effect of screenhouses on internal environmental parameters and tree physiology (Ferrarezi et al., 2017a; Ferrarezi et al., 2017b). Solar radiation and reference evapotranspiration were 22% and 23.8% lower inside the screenhouses compared to the open-air. Air temperature was greater inside the screenhouses whereas wind gusts were higher in the open-air. There was no difference in cumulative rainfall between both production systems (Ferrarezi et al., 2017a). Trees grown inside the enclosed screenhouses had greater canopy area compared to the open-air plots (Ferrarezi et al., 2017b). The larger canopy growth inside the screenhouses related to higher fruit yield and fruit quality. Schumann et al. (2018) reported yields greater than 76,218 kg/ha and with good internal quality and all met grade requirements for the fresh market (100% pack-out).

Our objectives were to assess the ability of enclosed screenhouses to exclude the ACP, stop HLB inoculation and dissemination, and improve fruit yield of in-ground and container-grown 6-year-old “Ray Ruby” grapefruit at super-high planting densities relative to open-air trees.

## Material and Methods

### Site Location

The study was established in Nov. 2013 at the UF/IFAS Indian River Research and Education Center in Fort Pierce, FL (lat. 27°26’N, long. 80°26’W, 10 m elevation above sea level).

### Meteorological Variables

**Figure 1:**
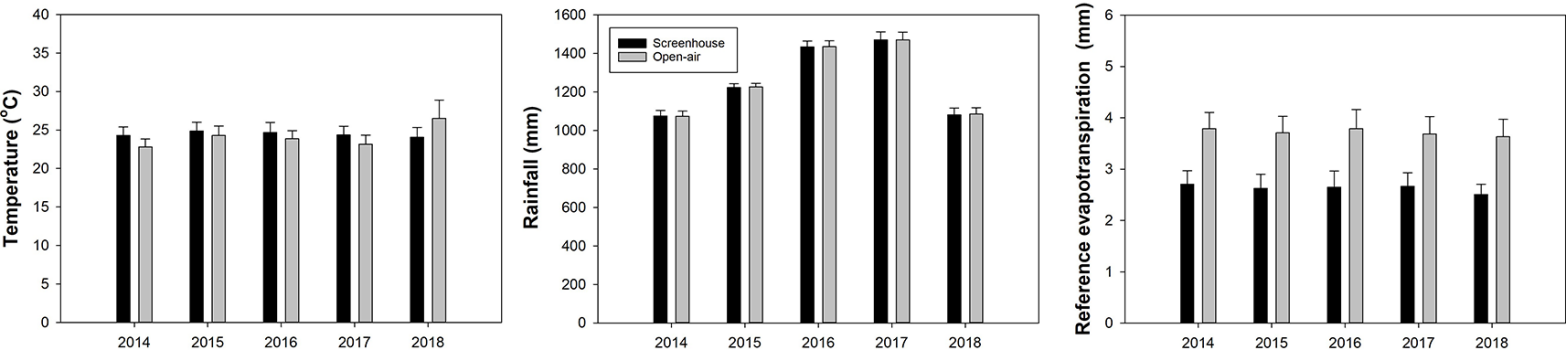
Air temperature, rainfall and reference evapotranspiration inside the screenhouse and in the open-air plots. Average of 365 days per year.

Two weather stations (WatchDog 2900ET; Spectrum Technologies, Aurora, IL) were installed inside two of the four screenhouses, and two stations were placed in two of the open-air control plots. All weather stations were mounted on wooden posts 2.15 m above ground. The two stations outside the screenhouses were placed 18 to 33 m away from their accompanying screenhouses to avoid any unwanted shading effects of the screenhouses on the weather stations. The straight-line distance separating the weather stations within the two individual screenhouses was 58 m, and the straight-line distance separating the weather stations in the two individual open-air plots was approximately 157 m. The weather stations recorded solar radiation, air temperature, wind gust, and rainfall every 30 min. Reference evapotranspiration (ET_o_) was calculated daily using the Penman-Monteith equation (Allen et al., 1998) (**Figure 1**). Solar radiation and wind gust data not shown.

### Treatments and Experimental Design

We tested two production systems (screenhouse and open-air), two planting systems (in-ground and potted), and two rootstocks {“Sour Orange” [*Citrus × aurantium*] and “US-897” [“Cleopatra” mandarin (*Citrus reticulata*) × “Flying Dragon” trifoliate orange (*Poncirus trifoliata*)]}, with four replications arranged in a randomized complete block split-split-plot experimental design. Production system was considered as the main plot, planting system as the split-plot and rootstock as the split-split-plot.

### Citrus Trees

“Ray Ruby” grapefruit trees were purchased from licensed, certified disease-free commercial nurseries (Sawmill Citrus Nursery, Fort Meade, FL and Brite Leaf, Lake Panasoffkee, FL). Trees were planted at a density of 1,957 trees/ha with spacing of 1.68 m in-row and 3.05 m between-row. Screenhouses contained four rows with eight trees each, while open-air plots contained three rows with eight trees each (total of 896 trees/0.46 ha). The same tree density was used in all treatments.

Potted trees were planted in 37.85-L plastic containers (#10 Accelerator AP-10; Nursery Supplies, Chambersburg, PA). The plastic containers were filled with a medium consisting (v/v) of 50% clean, washed silica sand, 15% Florida peat moss, 7.5% coconut fiber, 20% cypress sawdust, and 7.5% perlite (Harrell’s, Lake Placid, FL). Plastic growing containers were placed upon 103-cm^2^ ceramic tiles to prevent tree roots from growing into the underlying native soil.

In-ground trees were planted in Pineda soil series, classified as loamy, siliceous, active hyperthermic Arenic Glossaqualfs. This is very deep, nearly leveled with natural slopes ranging from 0 to 2%, and poorly drained soil.

### Screenhouses

**Figure 2:**
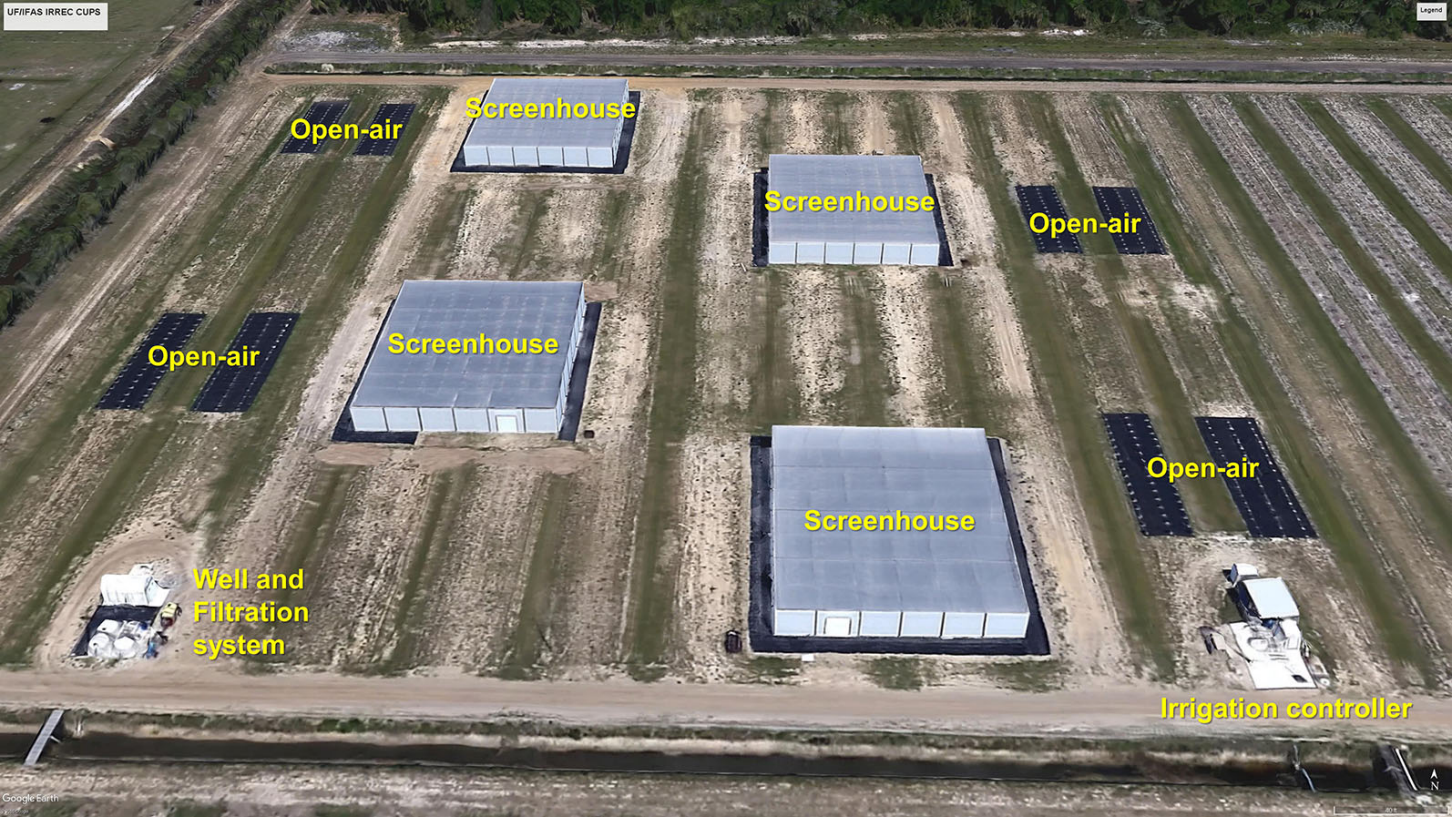
The University of Florida’s Institute of Food and Agricultural Sciences Indian River Research and Education Center citrus under protective screen has four passively ventilated 1,080-m^2^ completely enclosed screenhouses (30 m wide × 36 m long × 4.3 m tall) constructed using a 50-mesh monofilament high-density polyethylene (HDPE) screen (Signature Supply, Lakeland, FL). Source: Google Earth, July 2019. Credit: Rhuanito S. Ferrarezi.

Four passively ventilated 1,080-m^2^ completely enclosed screenhouses (30 m wide × 36 m long × 4.3 m tall) were constructed using a 50-mesh (0.297 mm) monofilament high-density polyethylene (HDPE) screen (Signature Supply, Lakeland, FL) (**Figure 2**). The main support for each enclosed, covered structure consisted of pressure-treated, wooden utility poles (Outdoor Living Products, Orlando, FL). Each main support utility pole was fixed to the ground with one guy-wire (1/4-inch-diameter, braided galvanized steel wire) and attached to two 5-ft-long earth anchors (Pierson Supply Company, Pierson, FL). Support utility poles located in the screenhouse corners were fixed to the ground with two guy-wires and four earth anchors (Ferrarezi et al., 2017a; Ferrarezi et al., 2017b).

The screen was attached to the sides of the structure by stapling the cloth to the interior side of the perimeter main support utility poles. The side screen cloth was attached to the top screen cloth with S-shaped galvanized steel hooks, and the side and top screen panels were pleated together, with the resulting seam directed toward the interior of the house. The construction of each screenhouse included one aluminum roll-up garage-style service door (2.44 m wide × 3.05 m tall). A 3.66-m wide × 3.66-m long × 3.66-m tall antechamber was built to limit insect inclusion when the entrance door is opened (Ferrarezi et al., 2017a; Ferrarezi et al., 2017b).

All four screenhouses and open-air plots were surrounded by a 15-m buffer area to prevent any influence on micrometeorological conditions to the next-nearest screenhouse and open-air plots.

The ground surface on both screenhouse and open-air plots were covered using a 3.66 m × 142 g weed fabric shade cloth (PRO-5 Weed-Barrier; DeWitt, Sikeston, MO) to reduce the weed population and facilitate weed control.

### Irrigation

Each tree in this trial was serviced by two 7.5-L/h flow drip emitters (SB-20; Bowsmith, Exeter, CA). Trees grown in screenhouses and open-air plots were watered to replenish the corresponding daily ET_o_ obtained from the weather stations for their respective production systems. Monthly total and cumulative ET_o_ values for screenhouses and open-air plots are provided in Ferrarezi et al. (2017a). From Jan. to July 2014, all trees automatically received daily irrigation volumes that were approximately 33% of the total ET_o_ due to lower water demand. From July to Dec. 2014 onwards, trees received daily irrigation volumes that were 100% of the total ET_o_. Trees were not irrigated on days where rainfall was equal to or greater than the ET_o_.

### Fertigation

We used a 15N-2.6P-22.4K water-soluble fertilizer (Agrolution pHLow; Everris NA, Dublin, OH) with 15% total-nitrogen (2.6% ammoniacal and 12.4% nitrate), 2.6% phosphorus (P), 22.4% potassium (K), 3.3% calcium (Ca), 0.02% boron (B), 0.05% copper (Cu), 0.1% iron (Fe), 0.05% manganese (Mn), 0.0005% molybdenum (Mo), and 0.05% zinc (Zn). Fertilizer was mixed at a concentration of 22.68 kg fertilizer/378.5 L water in a 1,893-L plastic stock tank plumbed in-line with the servicing irrigation system. A proportional 151.4 L/min chemical injector (D8RE2; Dosatron International, Clearwater, FL) was installed directly up-stream to the irrigation zone valves and connected to the fertigation stock tank. This injector added fertigation solution to each irrigation event and was adjusted seasonally to increase or decrease the proportional volume of fertigation solution added to the irrigation stream. The proportional injector’s settings changed over time based on nutritional needs by season, and the minimum, maximum, and annual mean of the proportioner (v/v) were: 0.2% (February), 1.9% (September), and 0.8%. The screenhouses and the open-air plots received the same amount of fertilizer throughout the study.

### Psyllid Monitoring

Psyllids were monitored monthly from planting until April 2018, when the scouting started bimonthly. We used 10 × 17.5 cm yellow sticky traps (Alpha Scents, West Linn, OR). The sticky cards were examined in the laboratory to search for adult psyllids.

### *Candidatus* Liberibacter Asiaticus Diagnostic

Citrus samples were screened yearly (2015-2018) or twice a year (2014 and 2019) for the presence of *C*Las. Ten fully expanded leaves and leaf petioles with intact stems from symptomatic branches (if present) were collected in eight trees per plot. Diagnostic analysis was done by the quantitative real-time polymerase chain reaction (qRT-PCR) tests coupled with U.S. Department of Agriculture’s Animal and Plant Health Inspection Service approved primers (Li et al., 2006) at the HLB Diagnostic Laboratory at the UF/IFAS Southwest Florida Research and Education Center in Immokalee, FL from 2014 to 2016 and at the Southern Gardens Diagnostic Laboratory in Clewiston, FL since 2017.

### Tree Canopy Growth Parameters

Tree canopy growth parameters were measured every 6 months. Eight trees were measured per screenhouse plot for each planting system and rootstock (total *n* = 64). Six trees were measured per open-air plot for each planting system and rootstock (total *n* = 48). Tree size was assessed every year by measuring trunk diameter (5 cm above the bud union), tree height to top of canopy (not including height of vigorous shoots that extend significantly past the top of the canopy), and canopy diameter (in parallel and perpendicular to the tree row). Canopy volume was calculated using the formula: [(diameter parallel to row × diameter perpendicular to row) × height] ÷ 4.

### Fruit Yield, Fruit Diameter, and Number of Fruit

Fruit yield was determined at maturity by strip-harvesting all fruit from eight trees per plot, weighing the amount of fruit picked per tree and running it through an optical sizer (Autoline, Reedley, CA) mounted on a trailer. The number of fruit per tree and fruit diameter were determined for all fruit harvested on each tree. We categorized fruit size as small (<100 mm), adequate (100–117 mm), and large (>117 mm) based on the commercial categories used to classify grapefruit (number of fruit per cartons): size 48 and smaller, size 40–27, and size 23 and larger, respectively.

### Soluble Solids Content, Titratable Acidity, Ratio, and Yield of Solids

Random samples of 20 fruit from each experimental unit were collected for fruit quality analysis. The fruit samples were weighed, and fruit diameter at the equator was measured with a digital caliper. The fruit were cut in half, and juiced using a press juicer (model 2702; Brown International Corp, Covina, CA); then, juice was weighed, and expressed as a percentage of the total fruit weight. Soluble solids content was determined with a temperature-compensated, digital refractometer (HI96801; Hanna Instruments, Woonsocket, RI) using a few drops of juice. The total acidity (percent anhydrous citric acid) was determined by titrating juice samples to pH 8.3 with NaOH using an automatic titrator (HI931; Hanna Instruments, Smithfield, RI). The empirical soluble solids content/acid ratio, calculated by dividing the soluble solids content by the titratable acidity, is one of the most commonly used indicators of juice quality (Kimbal, 1991). Yield of solids per hectare was calculated as: (% juice in fruit ÷ 100) × (soluble solids content ÷ 100) × fruit yield (kg/ha).

### Leaf Nutritional Status

Leaf samples were collected once a year. Six leaves per tree for eight trees per screenhouse on each planting system and rootstock, and six trees per open-air on each planting system and rootstock. Healthy, asymptomatic leaves on mature, hardened-off flushes were picked, washed in phosphate-free detergent, rinsed in distilled water, and placed into a drying oven at 50°C for 5 to 7 days. Dried leaves were sent to the UF/IFAS Analytical Services Laboratories in Gainesville, FL for determination of leaf N, Mg, Mn, Zn, and Fe and Waters Agricultural Lab in Camila, GA for determination of leaf macro and micronutrients.

### Statistical Analysis

We collected six or eight sub-samples from each experimental unit depending on the production system plot size. Data were averaged to provide a single sample value representing each replication (n = 4). All variables were analyzed using general linear model procedures in SAS (version 9.4; SAS Institute, Cary, NC). The errors were checked to be normally and independently distributed, and data were presented by eight treatment combinations. Probability values were considered statistically significant when P ≤ 0.05. Independent factor (production systems, planting systems and rootstocks) probability values are available in the **Supplementary Material (Data Sheet 1)**.

## Results and Discussion

**Figure 3:**
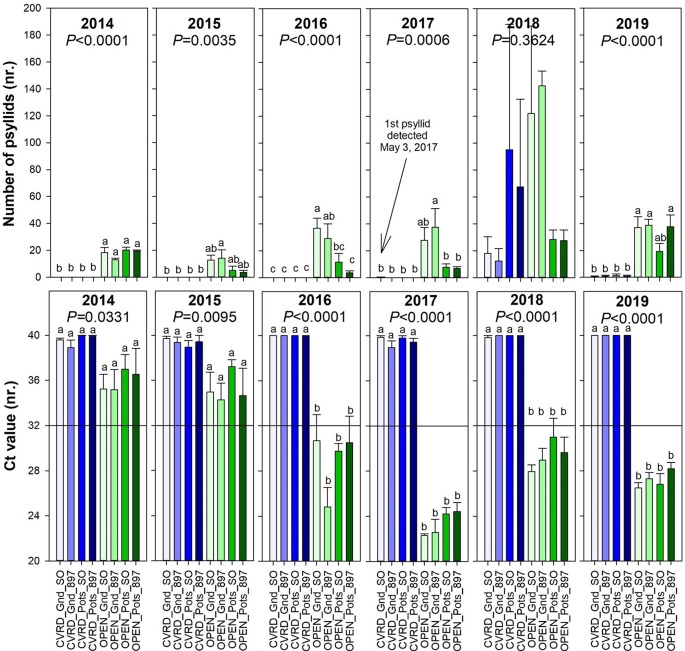
Number of psyllids (top) and cycle threshold (Ct) value of *Candidatus* Liberibacter asiaticus (*C*Las) deoxyribonucleic acid (DNA) (bottom) of “Ray Ruby” grapefruit trees cultivated under two production systems (screenhouse, CVRD and open-air, OPEN), two planting systems (in-ground, GND and potted, POTS), and two rootstocks (“Sour Orange,” SO, and “US-897”), with four replications arranged in a split-split-plot experimental design. Mean ± standard error of four replications. Means with the same letter are not significantly different from each other at 5% probability (*P ≤* 0.05) using Tukey mean comparison test. Reference line on bottom graph indicate the threshold to consider trees infected by *C*Las : < 32 negative and ≥ 32 positive.

The screenhouses are able to keep psyllids out when the screen is intact. No psyllids were detected prior to 2017 according to Ferrarezi et al. (2017a). However, the subsequent passage of hurricanes or tropical storms tore the screens, allowing psyllids to enter into the screenhouses. The first single psyllid inside the UF/IFAS Indian River Research and Education Center (IRREC) CUPS was detected in May 2017, 7 months after Hurricane Matthew caused minor damages to the screen in October 2016 (**Figure 3**, top). In September 2017, Hurricane Irma caused severe damages to all four screenhouses, by loosening guy wires, bending peripheral poles, lifting off weed ground cloth, overturning all open-air pots, lifting out center poles 30 to 60 cm of ground, causing rips in screens, and provoking the detachment of S hooks. Repairs were completed in April 2018, but large openings in the screenhouse allowed psyllids to enter the enclosures. Psyllid population increased quickly and were difficult to control due to a faulty sprayer, reaching on average 15.13 psyllids in treatments in-ground and 81.25 psyllids in pots when averaging the two rootstocks. During this time, there were no significant differences among any of the treatments (**Figure 3**, top, *P =* 0.3624). In 2019 ≈1 psyllid was detected in in-ground trees and 1 psyllid in pots due to another tropical storm in April 2019 (**Figure 3**, top). Despite the presence of psyllids in the screenhouses in 2018 (2017 and 2019 numbers are insignificant), HLB was never detected in any of the trees. (**Figure 3**, bottom, *P* = 0.0014) It is possible the psyllids that multiplied inside the screenhouses were not infected by the *C*Las and the lack of constant re-inoculation was sufficient to prevent infection to occur (**Figure 3**, bottom, *P =* 0.0014).

**Figure 4:**
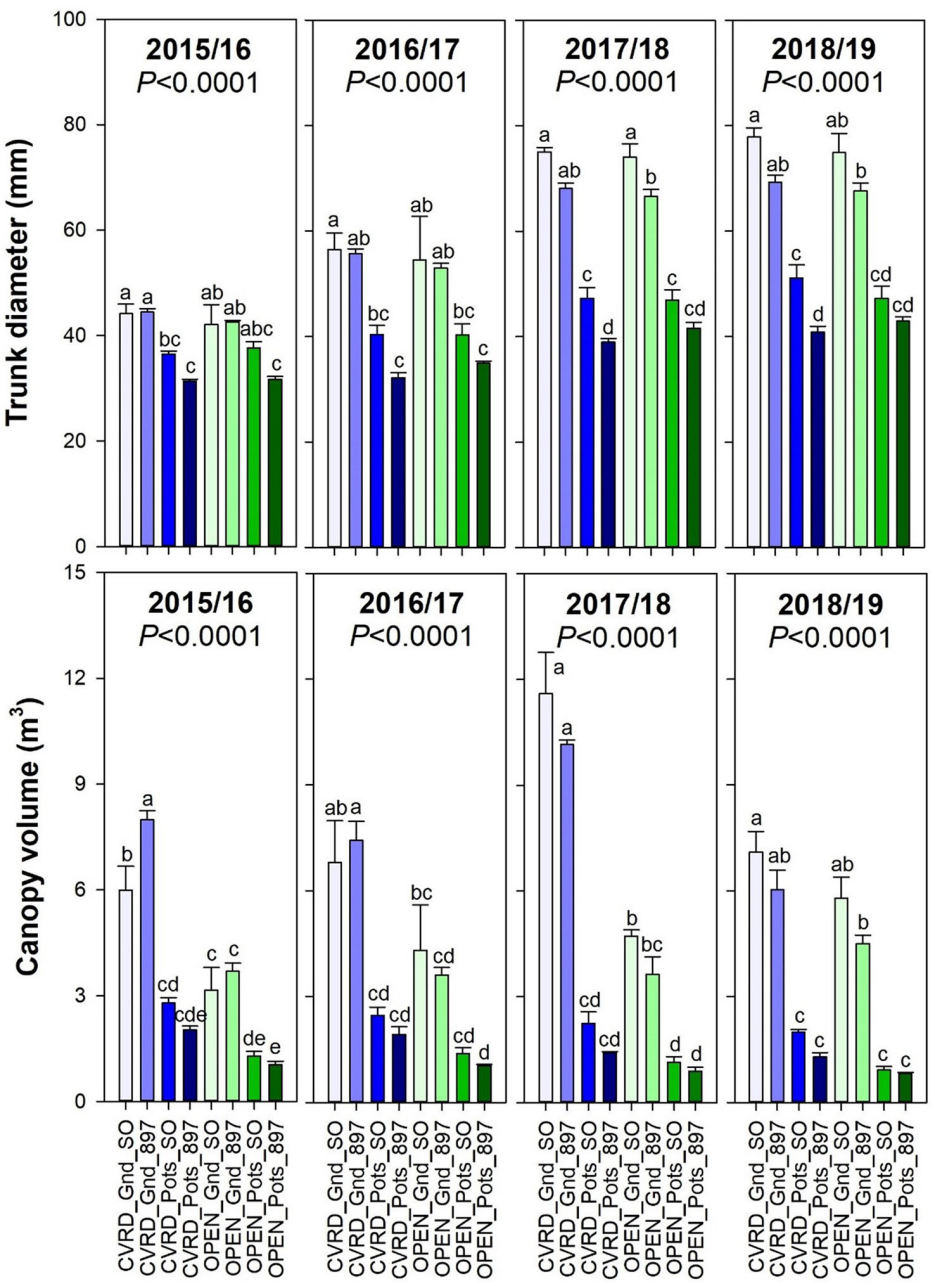
Trunk diameter (top) and canopy volume (bottom) of “Ray Ruby” grapefruit trees cultivated under two production systems (screenhouse, CVRD and open-air, OPEN), two planting systems (in-ground, GND and potted, POTS), and two rootstocks (“Sour Orange,” SO, and “US-897”), with four replications arranged in a split-split-plot experimental design. Mean ± standard error of four replications. Means with the same letter are not significantly different from each other at 5% probability (*P ≤* 0.05) using Tukey mean comparison test.

Trunk diameter increased overtime in all treatments as trees continued to grow, with in-ground trees showing the highest values (**Figure 4**, top, *P* < 0.0001). There were no significant differences between the screenhouse and open-air treatments. Canopy volume increased only in the in-ground treatments (**Figure 4**, bottom, *P* < 0.0001). In 2018/19 the low canopy volume value is the result of tree hedging and topping. Potted trees resulted in the lowest volume possibly due to the growing media used: a medium consisting mainly by washed silica sand, since all the organic matter initially added was decomposed after 2 years.

**Figure 5:**
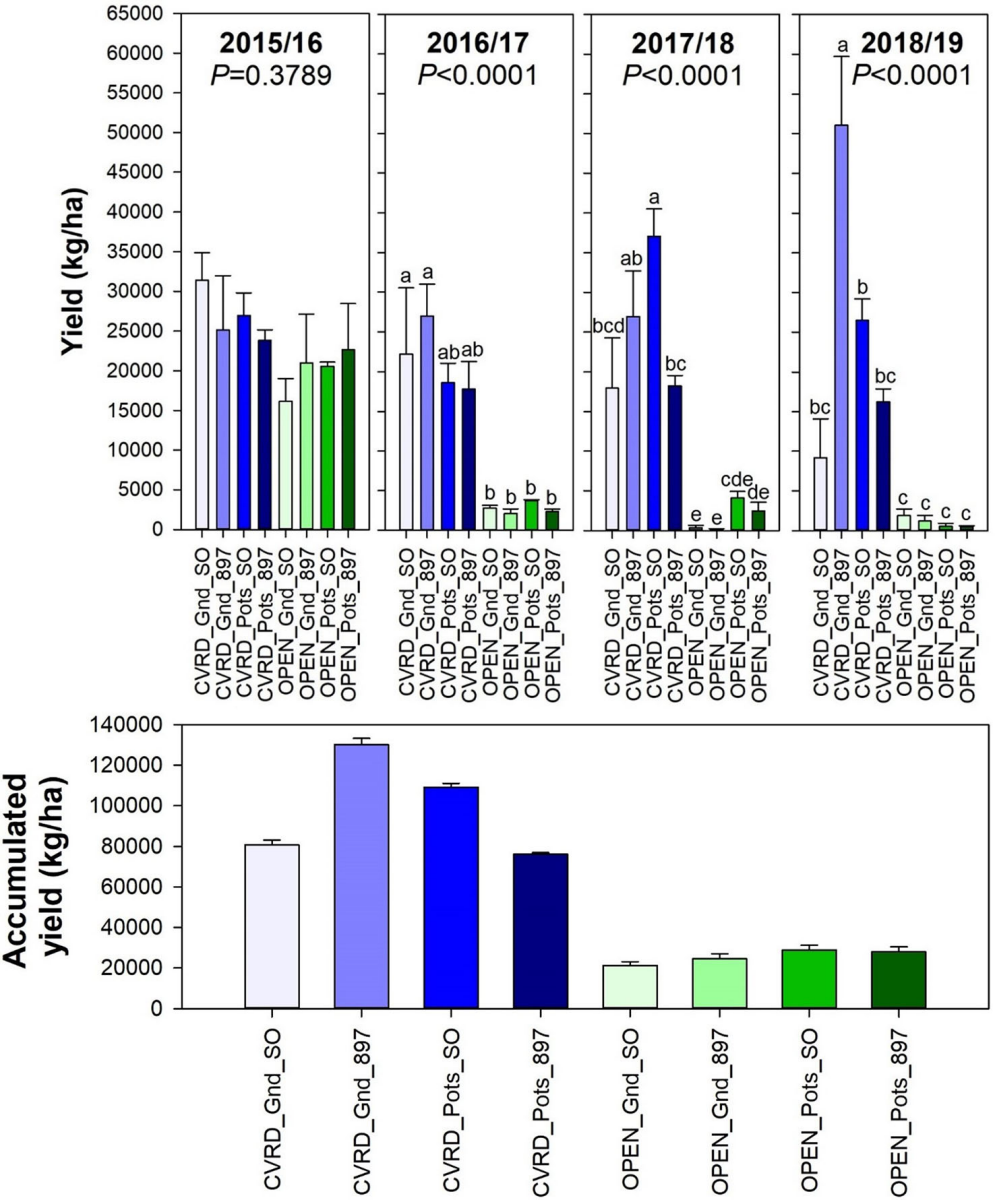
Fruit yield of “Ray Ruby” grapefruit trees cultivated under two production systems (screenhouse, CVRD and open-air, OPEN), two planting systems (in-ground, GND and potted, POTS), and two rootstocks (“Sour Orange,” SO, and “US-897”), with four replications arranged in a split-split-plot experimental design. Mean ± standard error of four replications. Means with the same letter are not significantly different from each other at 5% probability (*P ≤* 0.05) using Tukey mean comparison test.

Trees cultivated under screenhouses produced the greatest fruit yield. The treatment planted in-ground and on “US-897” yielded 51,081 kg/ha—twice the potted treatment on “Sour Orange” and thrice on “US-897” (**Figure 5**, *P* < 0.0001). This value is four times the 2017/18 U.S. average of 13,842 kg/ha (U.S. Department of Agriculture, 2019). Even though the fruit yield was higher than the national average, trees cultivated in pots did not perform as expected in this study. One of the justifications was the use of sand as a substrate. Over time, the organic matter decomposed, and the resulting media was 100% coarse sand. This reduced the water holding capacity and the water availability, most likely resulting in drought stresses that led to lower fruit yields. The accumulated fruit yield after 4 years was 130,306 kg/ha for trees grown under the screenhouse planted in-ground and on “US-897” followed by trees under the enclosures in pots on “Sour Orange” rootstock (**Figure 5**).

**Figure 6:**
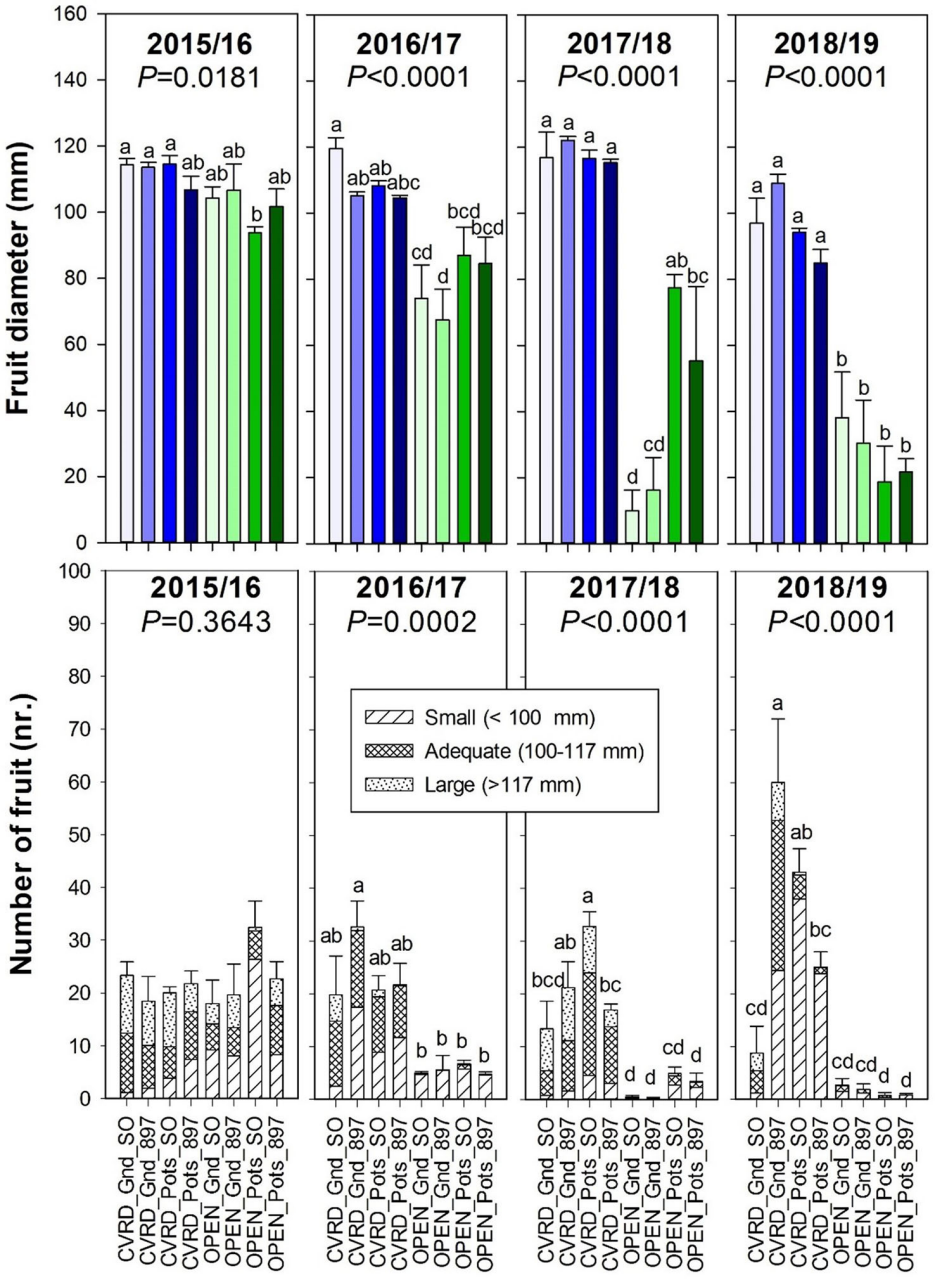
Fruit diameter (top) and number of fruit (bottom) of “Ray Ruby” grapefruit trees cultivated under two production systems (screenhouse, CVRD and open-air, OPEN), two planting systems (in-ground, GND and potted, POTS), and two rootstocks (“Sour Orange,” SO, and “US-897”), with four replications arranged in a split-split-plot experimental design. Mean ± standard error of four replications. Means with the same letter are not significantly different from each other at 5% probability (*P ≤* 0.05) using Tukey mean comparison test.

Fruit diameter and number of fruit on covered trees was statistically higher than the open-air treatments (**Figure 6**, *P* < 0.0001). The data represents the average of eight trees and four replications, and several trees in the open-air had zero fruit due to the negative effect of HLB disease. The number of fruit increased drastically in 2018/19 in comparison to previous years (**Figure 6**, bottom, *P* < 0.0001). That is expected since trees reached bearing age. However, there as a significant amount of small-sized fruit (<100 mm). Fruit diameter continued to decline with progressing years in open-air treatments, likely because of the high HLB incidence.

**Figure 7:**
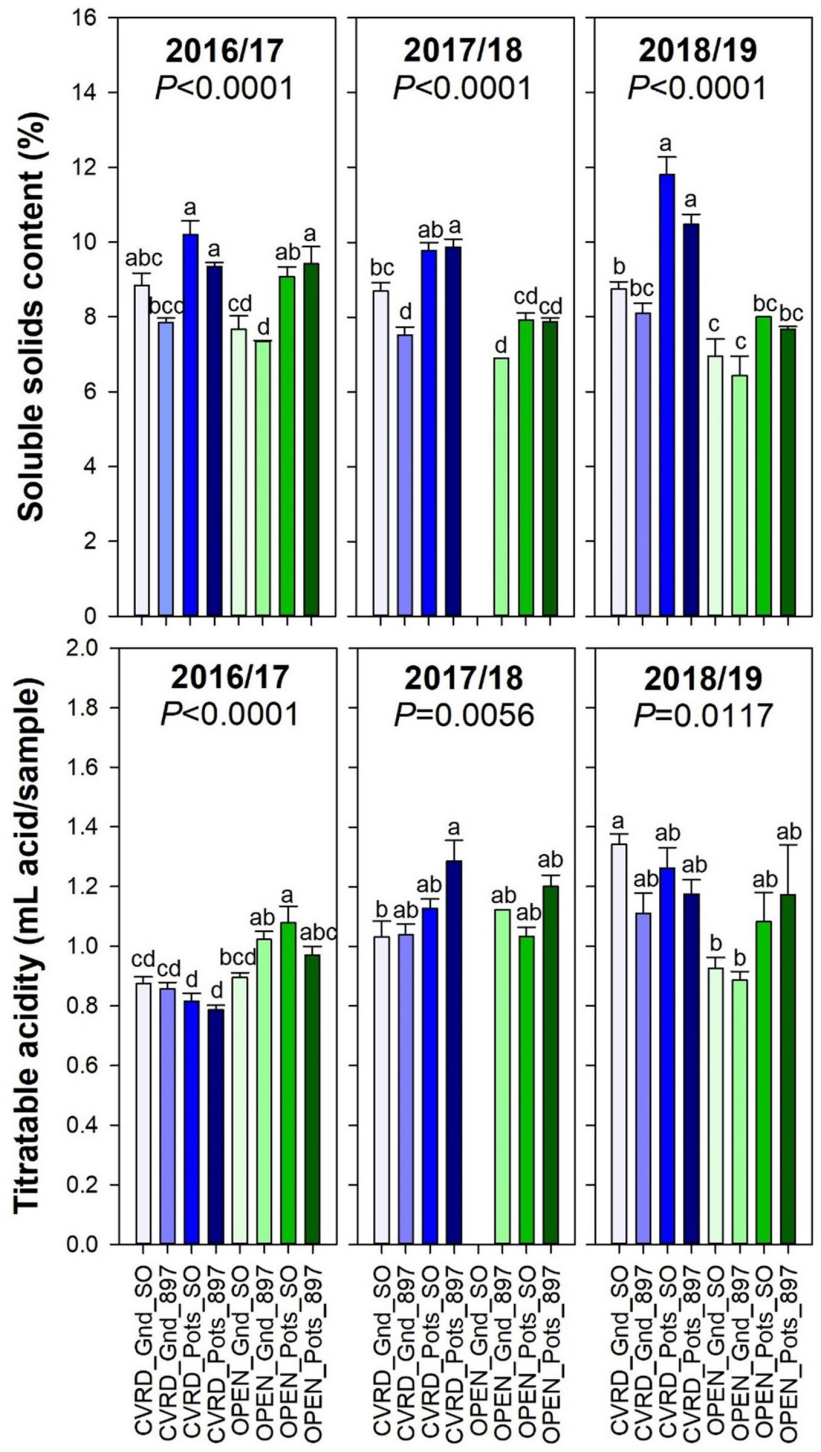
Soluble solids content (top) and titratable acidity (bottom) of “Ray Ruby” grapefruit trees cultivated under two production systems (screenhouse, CVRD and open-air, OPEN), two planting systems (in-ground, GND and potted, POTS), and two rootstocks (“Sour Orange,” SO, and “US-897”), with four replications arranged in a split-split-plot experimental design. Mean ± standard error of four replications. Means with the same letter are not significantly different from each other at 5% probability (*P ≤* 0.05) using Tukey mean comparison test.

The soluble solids content was high in potted trees with 11.8% in “Sour Orange” rootstock and 10.5% in “US-897” probably due to the constant stress induced by the growing media and limited container volume (**Figure 7**, top, *P* < 0.0001). In-ground trees in open-air resulted in the lowest soluble solids content and titratable acidity (**Figure 7**, bottom, *P* < 0.0117).

**Figure 8:**
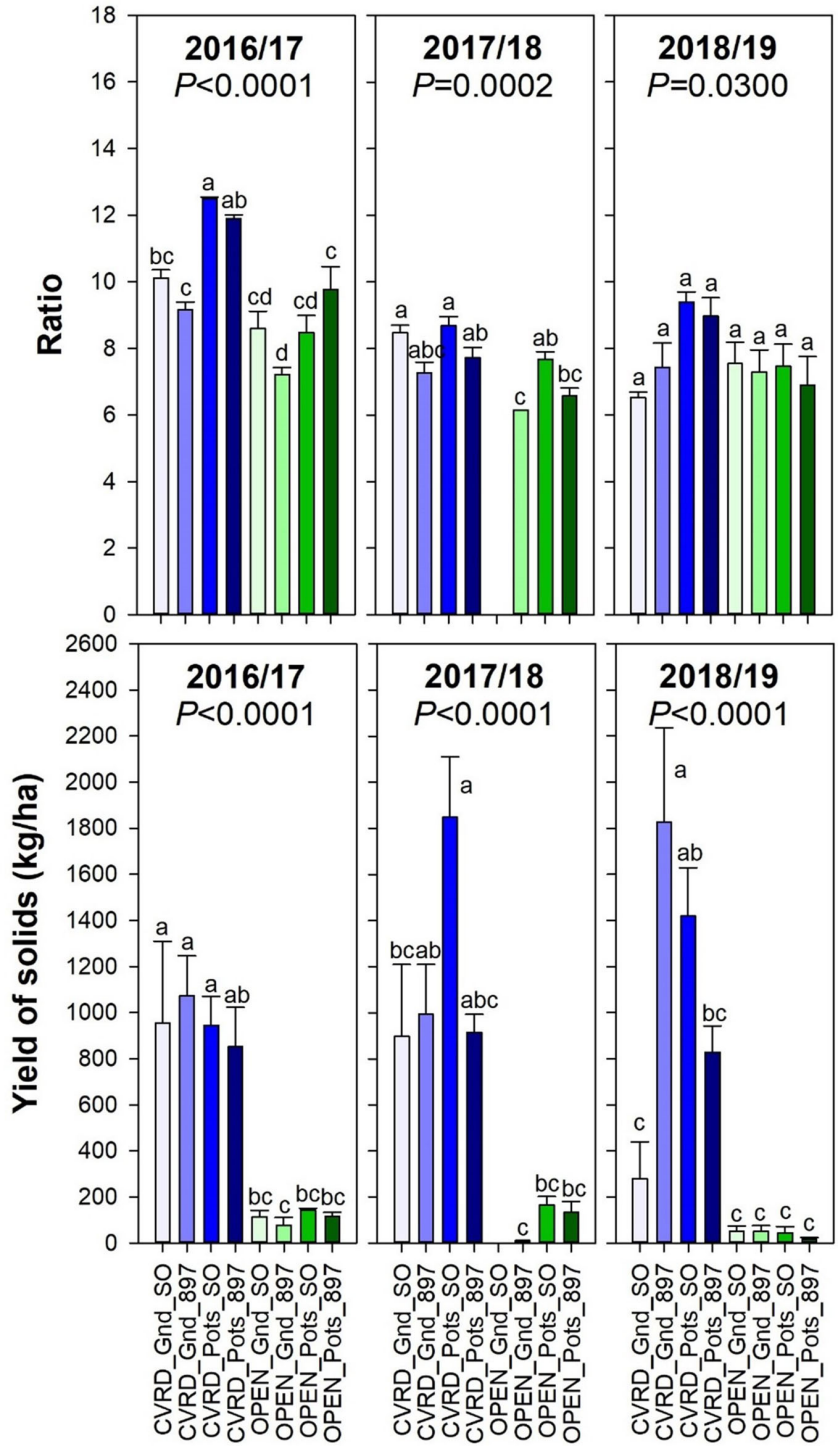
Soluble solids content: titratable acidity ratio (top) and Yield of solids (bottom) of “Ray Ruby” grapefruit trees cultivated under two production systems (screenhouse, CVRD and open-air, OPEN), two planting systems (in-ground, GND and potted, POTS), and two rootstocks (“Sour Orange,” SO, and “US-897”), with four replications arranged in a split-split-plot experimental design. Mean ± standard error of four replications. Means with the same letter are not significantly different from each other at 5% probability (*P ≤* 0.05) using Tukey mean comparison test. Empty bar on OPEN_Gnd_SO treatment in 2017/18 is related to the absence of fruit for fruit quality sampling in all reps.

In Florida, the grapefruit harvested for the fresh fruit market needs a ratio of at least 8:1 or 8. This value changes with titrabable acidity and time of the year, and is just an industry reference. The average ratio in the 2018/19 season was approximately 7.7 (**Figure 8**, top, *P* < 0.03). Ratio declined over time, but acids tended to increase. Yield of solids represents the amount of juice per box of citrus by the soluble solids content, indicating the economic return to growers. If all yield for fresh fruit production is converted into yield of solids per hectare (not a common practice in the fresh fruit market; used for reference here), the highest amount was obtained in covered trees planted in-ground on “US-897” (1,826 kg solids/ha) and in pots on “Sour Orange” (1,419 kg solids/ha) (**Figure 8**, bottom, *P* < 0.0001).

Nutrient content in leaf tissue varied by year as the trees grew larger (**Tables 1** and **2**, *P* < 0.05). Soon after planting from 2014 through 2016, treatment differences in leaf nutrient concentrations were observed as a result of in-ground *vs.* container-grown, and screenhouse *vs.* open-air treatments. However, no treatment effects of leaf nutrient concentrations were observed over time as tree developed. Throughout the experiment, both rootstocks exhibited similar leaf macronutrient and micronutrient concentrations. In the early stages of the experiment (2014 through 2016), container-grown trees tended to exhibit higher leaf N, P, and K concentrations than in-ground trees, but by 2017 these macronutrients concentrations equilibrated between treatments (**Table 1**, *P* < 0.05). However, this result did not extend to the micronutrient concentrations as there was no major container *vs*. in-ground effect. Overall, there was not effect of screenhouse *vs*. open-air treatments on either macronutrient or micronutrient concentrations. For the most part, micronutrient concentrations were seldom affected by any treatment (**Table 2**, *P* < 0.05).

**Table 1:** Leaf macronutrient content of “Ray Ruby” grapefruit trees cultivated under two production systems (screenhouse and open-air), two planting systems (in-ground and potted), and two rootstocks (“Sour Orange,” SO, and “US-897”), with four replications arranged in a split-split-plot experimental design. Mean ± standard error of four replications.

	N (%)								P (%)							
Year	Screenhouse				Open-air				Screenhouse				Open-air			
	In-ground		Potted		In-ground		Potted		In-ground		Potted		In-ground		Potted	
	SO	US-897	SO	US-897	SO	US-897	SO	US-897	SO	US-897	SO	US-897	SO	US-897	SO	US-897
**2014**	2.3 c	2.3 c	2.8 b	2.8 b	2.7 b	2.7 b	3.2 a	3.1 a	0.1 d	0.1 cd	0.2 bc	0.1 bcd	0.2 abc	0.2 ab	0.2 a	0.2 a
**2015**	2.7 d	2.6 d	3.5 bc	3.9 a	2.9 d	2.8 d	3.4 c	3.8 ab	0.2 c	0.2 c	0.2 a	0.2 a	0.2 c	0.2 bc	0.2 ab	0.2 ab
**2016**	2.3 a	2.4 a	2.4 a	2.4 a	2.3 a	2.5 a	2.6 a	2.6 a	0.2 abc	0.3 a	0.2 bc	0.2 c	0.2 bc	0.2 ab	0.2 bc	0.2 bc
**2017**	3.0 c	3.0 c	3.1 bc	3.1 bc	3.1 bc	3.2 abc	3.5 ab	3.6 a	0.2 a	0.2 a	0.2 a	0.2 a	0.2 a	0.2 a	0.2 a	0.2 a
**2018**	1.9 c	2.1 bc	2.1 abc	2.1 bc	2.2 abc	2.5 abc	2.7 a	2.6 ab	0.2 a	0.2 a	0.2 a	0.2 a	0.2 a	0.2 a	0.2 a	0.2 a
																
	**K (%)**								**Ca (%)**							
**Year**	**Screenhouse**				**Open-air**				**Screenhouse**				**Open-air**			
	**In-ground**		**Potted**		**In-ground**		**Potted**		**In-ground**		**Potted**		**In-ground**		**Potted**	
	**SO**	**US-897**	**SO**	**US-897**	**SO**	**US-897**	**SO**	**US-897**	**SO**	**US-897**	**SO**	**US-897**	**SO**	**US-897**	**SO**	**US-897**
**2014**	2.5 c	2.2 c	3.9 a	3.8 ab	2.5 c	2.2 c	3.5 ab	3.3 b	3.0 bc	3.8 a	1.9 d	2.0 d	2.9 c	3.6 ab	1.9 d	2.5 cd
**2015**	3.2 c	2.4 d	4.5 a	4.2 a	2.5 d	2.1 d	3.7 b	3.6 bc	2.6 bc	3.6 a	1.9 d	2.2 cd	2.3 cd	3.1 ab	2.0 d	2.3 cd
**2016**	2.3 a	2.3 a	2.0 ab	1.8 bc	1.6 cd	1.4 d	1.6 cd	1.6 bcd	4.2 abc	4.5 abc	4.5 ab	4.8 a	4.4 abc	4.0 bc	3.8 c	4.3 abc
**2017**	2.9 ab	2.6 ab	3.0 a	3.3 a	2.7 ab	2.3 b	2.7 ab	2.9 ab	3.6 abc	4.1 a	3.8 cb	3.8 ab	2.8 c	2.9 bc	2.7 c	2.7 c
**2018**	2.3 a	2.2 a	1.8 a	1.9 a	1.8 a	1.7 a	1.9 a	1.8 a	4.7 ab	5.0 ab	5.4 a	5.2 ab	4.6 ab	4.3 b	4.2 b	4.2 b
																
	**Mg (%)**								**S (%)**							
**Year**	**Screenhouse**				**Open-air**				**Screenhouse**				**Open-air**			
	**In-ground**		**Potted**		**In-ground**		**Potted**		**In-ground**		**Potted**		**In-ground**		**Potted**	
	**SO**	**US-897**	**SO**	**US-897**	**SO**	**US-897**	**SO**	**US-897**	**SO**	**US-897**	**SO**	**US-897**	**SO**	**US-897**	**SO**	**US-897**
**2014**	0.2 abc	0.2 bcd	0.1 cd	0.1 d	0.2 ab	0.2 a	0.2 bcd	0.2 bcd	0.3 a	0.3 a	0.3 a	0.3 a	0.3 a	0.3 a	0.3 a	0.3 a
**2015**	0.1 b	0.1 ab	0.1 c	0.1 c	0.2 ab	0.2 a	0.1 c	0.1 c	N/A	N/A	N/A	N/A	N/A	N/A	N/A	N/A
**2016**	0.3 ab	0.3 a	0.3 ab	0.3 a	0.2 bc	0.3 abc	0.2 c	0.2 bc	0.2 ab	0.3 a	0.2 ab	0.2 b	0.2 ab	0.2 ab	0.2 b	0.2 ab
**2017**	0.3 ab	0.3 a	0.3 abc	0.3 a	0.2 bc	0.2 abc	0.2 c	0.2 c	0.3 ab	0.3 ab	0.3 ab	0.3 a	0.3 b	0.3 b	0.3 ab	0.3 ab
**2018**	0.3 a	0.3 abc	0.2 ab	0.3 abc	0.2 bc	0.2 c	0.2 c	0.2 c	0.3 a	0.3 a	0.3 ab	0.3 a	0.3 ab	0.3 ab	0.3 ab	0.3 ab

Means with the same letter are not significantly different from each other at 5% probability (P ≤ 0.05) using Tukey mean comparison test.

**Table 2:** Leaf micronutrient content of “Ray Ruby” grapefruit trees cultivated under two production systems (screenhouse and open-air), two planting systems (in-ground and potted), and two rootstocks (“Sour Orange,” SO, and “US-897”), with four replications arranged in a split-split-plot experimental design.

	B (ppm)								Cu (ppm)							
Year	Screenhouse				Open-air				Screenhouse				Open-air			
	**In-ground**		**Potted**		**In-ground**		**Potted**		**In-ground**		**Potted**		**In-ground**		**Potted**	
	**SO**	**US-897**	**SO**	**US-897**	**SO**	**US-897**	**SO**	**US-897**	**SO**	**US-897**	**SO**	**US-897**	**SO**	**US-897**	**SO**	**US-897**
**2014**	94.4 a	94.6 a	84.8 a	88.7 a	79.4 a	78.5 a	75.4 a	78.5 a	97.4 a	93.1 a	76.1 a	77.0 a	86.0 a	83.5 a	86.8 a	84.9 a
**2015**	75.5 ab	79.6 a	72.9 ab	84.0 a	46.5 c	49.8 c	66.8 b	65.4 b	45.3 a	51.4 a	23.8 b	26.8 b	55.9 a	51.7 a	51.1 a	45.3 a
**2016**	82.7 ab	86.4 ab	85.3 ab	91.5 ab	78.4 ab	67.1 b	80.9 ab	98.2 a	39.9 bc	30.8 c	32.8 c	42.0 bc	97.6 ab	98.1 ab	130.3 a	107.0 a
**2017**	75.3 abc	77.2 abc	86.1 ab	90.1 a	60.6 c	56.5 c	65.8 bc	70.3 abc	134.9 a	141.0 a	139.9 a	119.2 a	82.7 a	84.0 a	91.9 a	94.4 a
**2018**	84.9 a	89.3 a	90.7 a	97.6 a	79.9 a	72.0 a	75.9 a	81.7 a	127.1 a	152.0 a	137.1 a	131.9 a	101.0 a	107.6 a	103.2 a	114.5 a
																
	**Fe (ppm)**								**Mn (ppm)**							
**Year**	**Screenhouse**				**Open-air**				**Screenhouse**				**Open-air**			
	**In-ground**		**Potted**		**In-ground**		**Potted**		**In-ground**		**Potted**		**In-ground**		**Potted**	
	**SO**	**US-897**	**SO**	**US-897**	**SO**	**US-897**	**SO**	**US-897**	**SO**	**US-897**	**SO**	**US-897**	**SO**	**US-897**	**SO**	**US-897**
**2014**	57.1 c	70.7 bc	76.3 abc	70.4 bc	74.9 abc	97.0 ab	107.0 a	82.3 abc	99.0 a	85.7 a	78.0 a	84.0 a	104.9 a	109.0 a	100.9 a	102.4 a
**2015**	58.9 ab	59.9 ab	66.2 ab	50.5 ab	49.7 b	61.8 ab	80.5 a	61.5 ab	39.8 a	36.4 a	31.1 a	29.8 a	27.4 a	32.3 a	32.2 a	29.3 a
**2016**	58.9 ab	73.1 a	64.3 ab	46.9 b	64.2 ab	74.6 a	61.9 ab	51.8 ab	17.5 c	20.1 bc	16.5 c	16.0 c	22.8 abc	32.0 a	29.5 ab	25.3 abc
**2017**	86.5 a	95.6 a	77.8 a	77.3 a	70.6 a	77.0 a	70.6 a	66.4 a	20.6 a	21.3 a	19.2 a	20.8 a	17.4 a	22.9 a	20.1 a	19.9 a
**2018**	86.5 a	99.8 a	88.1 a	82.5 a	58.9 a	71.6 a	60.6 a	49.0 a	20.1 ab	22.5 a	19.3 ab	21.7 a	16.3 ab	20.2 ab	17.1 ab	13.5 b
																
	**Zn (ppm)**															
**Year**	**Screenhouse**				**Open-air**											
	**In-ground**		**Potted**		**In-ground**		**Potted**									
	**SO**	**US-897**	**SO**	**US-897**	**SO**	**US-897**	**SO**	**US-897**								
**2014**	32.0 c	30.2 c	33.2 bc	32.9 bc	40.8 ab	42.3 a	44.3 a	42.0 a								
**2015**	18.7 ab	18.8 ab	21.2 a	16.5 b	18.0 ab	19.0 ab	21.8 a	20.8 a								
**2016**	26.8 cd	23.5 d	26.6 cd	33.4 bcd	38.5 abc	37.6 abc	44.1 ab	49.5 a								
**2017**	24.2 a	26.1 a	26.0 a	27.3 a	25.7 a	28.2 a	28.9 a	31.2 a								
**2018**	20.8 a	19.9 a	19.7 a	23.5 a	23.6 a	22.9 a	24.6 a	25.1 a								

Mean ± standard error of four replications. Means with the same letter are not significantly different from each other at 5% probability (P ≤ 0.05) using Tukey mean comparison test.

Ongoing studies in FL had shown horticultural feasibility in producing trees indoors due to high fruit yields and pack-outs. The economics of citrus undercover production systems is still being determined (Schumann and Singerman, 2016). The higher cost of CUPS must be offset by the highest possible yield of premium quality fresh fruit with a high market price in order to be profitable. Fortunately, the price of fresh fruit, especially for tangerine varieties, has been on an upward trend in recent years, totally justifying the investment cost. Because CUPS is a relatively new citrus production system with new challenges, current guidelines are preliminary and undergoing constant refinement through research. Mites and thrips may selectively enter through the permeable screen, while some of the larger beneficial pest predators are excluded. Greasy spot and other fungal diseases also thrive in the more humid conditions of the screenhouse environment. These non-lethal but economically important pests and diseases must be adequately controlled with integrated pest management approaches customized for CUPS in order to avoid loss of fruit yield and fruit quality.

However, these potential positive indicators are irrelevant if the screenhouse structure cannot resist high wind gusts and precipitation, and if the materials and design used to build large-scale facilities are not engineered to last and endure the weather. Most of the existing experimental and commercial facilities have been designed with limited engineering criteria, and the future of commercial CUPS in Florida lies on finding the adequate structure to resist extreme weather events since that is the highest percentage of the system initial investment.

## Conclusions

The screenhouses drastically reduced the number of psyllids in the citrus trees and avoided HLB disease, resulting in increased fruit yield and fruit quality. However, the screen effectiveness in blocking psyllids is susceptible to extreme weather events in Florida. The cost of the technology is still under evaluation along with structural modifications needed to deal with environmental challenges such as tropical storms and hurricanes. IRREC CUPS screenhouses are 6-years old, and the screen is experiencing degradation from ultraviolet light, rainfall, and wind, resulting in screen rupture at several points in the roof, requiring replacement since the material lifespan is over.

## Data Availability Statement

All datasets generated for this study are included in the article/**Supplementary Material**.

## Author Contributions

RF, JQ, AW, MR, and NM contributed to the data collection, analysis, writing and review of the manuscript.

## Funding

Funding for this research was provided by the University of Florida’s Institute of Food and Agricultural Sciences (UF/IFAS) Citrus Initiative, U. S. Department of Agriculture’s Florida Department of Agriculture and Consumer Services (FDACS) Specialty Crop Block Grant (SCBG) (project #00092195), U. S. Department of Agriculture's Specialty Crop Research Initiative (SCRI) Citrus Disease Research and Extension Program (CDRE) (Award #2018-70016-27387), and UF/IFAS IRREC’s Center Director Dr. Ronald Cave.

## Disclaimer

Mention of a trademark or proprietary product is for identification only and does not imply a guarantee or warranty of the product by the authors.

## Conflict of Interest

The authors declare that the research was conducted in the absence of any commercial or financial relationships that could be construed as a potential conflict of interest.
