# Supplementary material for: Citrus Production Under Screen as a Strategy to Protect Grapefruit Trees From Huanglongbing Disease
Source: Front Plant Sci. 2019 Dec 18;10:1598. doi: 10.3389/fpls.2019.01598 (PMC6930270; doi:10.3389/fpls.2019.01598)
Supplement: Supplementary file 1 [file DataSheet_1.pdf]

Citrus production under screen as a strategy to protect grapefruit trees from huanglongbing disease

Rhuanito S. Ferrarezi[1]*†, Jawwad A. Qureshi[2], Alan L. Wright[1], Mark A. Ritenour[1], and Natalia P. F. Macan[1]

[1]Indian River Research and Education Center, Institute of Food and Agricultural Sciences, University of Florida, Fort Pierce, FL, United States.

[2]Southwest Florida Research and Education Center, Institute of Food and Agricultural Sciences, University of Florida, Immokalee, FL, United States.

***Correspondence:** Rhuanito S. Ferrarezi, rferrarezi@ufl.edu

†Present Address: Rhuanito S. Ferrarezi, Indian River Research and Education Center, Institute of Food and Agricultural Sciences, University of Florida, Fort Pierce, FL, United States.

Citation: Ferrarezi RS, Qureshi JA, Wright AL, Ritenour MA and Macan NPF (2019) Citrus Production Under Screen as a Strategy to Protect Grapefruit Trees From Huanglongbing Disease. Front. Plant Sci. 10:1598. doi: 10.3389/fpls.2019.01598.

Supplementary materials (Tables with statistical analysis)

Probability (P) values for Number of Psyllids of 'Ray Ruby' grapefruit trees cultivated under two production systems (**ProdSys**; screenhouse and open-air), two planting systems (**Planting**; in-ground and potted), and two rootstocks (**Rootstock**; Sour orange and US-897).

**Source**	**2014**	**2015**	**2016**	**2017**	**2018**	**2019**
Model	*<0.0001*	*0.0035*	*<0.0001*	*0.0006*	*0.3624*	*<0.0001*
**ProdSys**	<0.0001	0.0002	<0.0001	0.0001	0.3471	<0.0001
**Planting**	0.0751	0.0325	0.0024	0.0068	0.5703	0.2143
**ProdSys*Planting**	0.0751	0.0325	0.0024	0.0073	0.0170	0.1797
**Rootstock**	0.2015	0.9755	0.3034	0.6264	0.9200	0.1692
**ProdSys*Rootstock**	0.2015	0.9755	0.3034	0.6064	0.6937	0.1592
**Planting*Rootstock**	0.3345	0.7353	0.9868	0.5482	0.7492	0.2681
**ProdSys*Planting*Rootstock**	0.3345	0.7353	0.9868	0.5294	0.9970	0.2269

Probability (P) values for Ct_value of *C*Las DNA of 'Ray Ruby' grapefruit trees cultivated under two production systems (**ProdSys**; screenhouse and open-air), two planting systems (**Planting**; in-ground and potted), and two rootstocks (**Rootstock**; Sour orange and US-897).

Source	2014	2015	2016	2017	2018	2019
Model	*0.0427*	*0.0126*	*<0.0001*	*<0.0001*	*<0.0001*	*<0.0001*
**ProdSys**	0.0004	<0.0001	<0.0001	<0.0001	<0.0001	<0.0001
**Planting**	0.8043	0.6586	0.7689	0.0196	0.1290	0.3939
**ProdSys*Planting**	0.7347	0.8282	0.7689	0.0556	0.1632	0.3939
**Rootstock**	0.4780	0.8967	0.1602	0.6522	0.9368	0.1290
**ProdSys*Rootstock**	0.6703	0.7831	0.1602	0.3064	0.8324	0.1290
**Planting*Rootstock**	0.8419	0.7252	0.3625	0.7947	0.3147	0.6888
**ProdSys*Planting*Rootstock**	0.6380	0.6893	0.3625	0.7145	0.3807	0.6888

Probability (P) values for HLB incidence of 'Ray Ruby' grapefruit trees cultivated under two production systems (**ProdSys**; screenhouse and open-air), two planting systems (**Planting**; in-ground and potted), and two rootstocks (**Rootstock**; Sour orange and US-897).

Source	2014	2015	2016	2017	2018	2019
Model	*0.0097*	*0.0014*	*<0.0001*	*<0.0001*	*<0.0001*	<0.0001
**ProdSys**	<0.0001	<0.0001	<0.0001	<0.0001	<0.0001	<0.0001
**Planting**	0.4056	0.3513	0.4233	.	0.3273	.
**ProdSys*Planting**	0.4056	0.3513	0.4233	.	0.3273	.
**Rootstoc**	0.9259	0.3513	0.4233	.	1.000	.
**ProdSys*Rootstoc**	0.9259	0.3513	0.4233	.	1.000	.
**Planting*Rootstoc**	0.7801	0.5738	0.1555	.	0.3273	.
**ProdSy*Planti*Rootst**	0.7801	0.5738	0.1555	.	0.3273	.

Probability (P) values for Trunk diameter of 'Ray Ruby' grapefruit trees cultivated under two production systems (**ProdSys**; screenhouse and open-air), two planting systems (**Planting**; in-ground and potted), and two rootstocks (**Rootstock**; Sour orange and US-897).

Source	2015	2016	2017	2018
Model	*<0.0001*	*<0.0001*	*<0.0001*	*<0.0001*
**ProdSys**	0.6013	0.8260	0.9587	0.2831
**Planting**	<0.0001	<0.0001	<0.0001	<0.0001
**ProdSys*Planting**	0.2393	0.4435	0.2859	0.6256
**Rootstock**	0.0305	0.1050	<0.0001	<0.0001
**ProdSys*Rootstock**	0.8954	0.8260	0.5809	0.2164
**Planting*Rootstock**	0.0140	0.2415	0.8728	0.8334
**ProdSys*Planting*Rootstock**	0.8150	0.7158	0.4355	0.4456

Probability (P) values for Canopy Volume of 'Ray Ruby' grapefruit trees cultivated under two production systems (**ProdSys**; screenhouse and open-air), two planting systems (**Planting**; in-ground and potted), and two rootstocks (**Rootstock**; Sour orange and US-897).

**Source**	**2015**	**2016**	**2017**	**2018**
Model	*<0.0001*	*<0.0001*	*<0.0001*	*<0.0001*
**ProdSys**	<0.0001	0.0002	<0.0001	0.0003
**Planting**	<0.0001	<0.0001	<0.0001	<0.0001
**ProdSys*Planting**	0.0001	0.0299	<0.0001	0.2286
**Rootstock**	0.1529	0.6164	0.0131	0.0059
**ProdSys*Rootstock**	0.3700	0.5518	0.4929	0.7173
**Planting*Rootstock**	0.0021	0.6680	0.2969	0.1478
**ProdSys*Planting*Rootstock**	0.0650	0.4199	0.8595	0.4484

Probability (P) values for Fruit Yield of 'Ray Ruby' grapefruit trees cultivated under two production systems (**ProdSys**; screenhouse and open-air), two planting systems (**Planting**; in-ground and potted), and two rootstocks (**Rootstock**; Sour orange and US-897).

**Source**	**2015**	**2016**	**2017**	**2018**
Model	*0.3789*	*<0.0001*	*<0.0001*	*<0.0001*
**ProdSys**	0.0368	<0.0001	<0.0001	<0.0001
**Planting**	0.9834	0.2680	0.0940	0.0742
**ProdSys*Planting**	0.3410	0.1856	0.6487	0.1549
**Rootstock**	0.8433	0.8568	0.2260	0.0072
**ProdSys*Rootstock**	0.1936	0.5637	0.4084	0.0051
**Planting*Rootstock**	0.9792	0.5413	0.0048	<0.0001
**ProdSys*Planting*Rootstock**	0.6362	0.6376	0.0098	<0.0001

Probability (P) values for Fruit diameter of 'Ray Ruby' grapefruit trees cultivated under two production systems (**ProdSys**; screenhouse and open-air), two planting systems (**Planting**; in-ground and potted), and two rootstocks (**Rootstock**; Sour orange and US-897).

**Source**	**2015**	**2016**	**2017**	**2018**
Model	*0.0181*	*<0.0001*	*<0.0001*	*<0.0001*
**ProdSys**	0.0012	<0.0001	<0.0001	<0.0001
**Planting**	0.0689	0.3323	0.0011	0.0314
**ProdSys*Planting**	0.4585	0.0327	0.0003	0.9603
**Rootstock**	0.8711	0.1566	0.6631	0.9320
**ProdSys*Rootstock**	0.1165	0.6342	0.4719	0.7638
**Planting*Rootstock**	0.9084	0.4328	0.2082	0.6617
**ProdSys*Planting*Rootstock**	0.2936	0.7212	0.4249	0.1975

Probability (P) values for Number of fruit of 'Ray Ruby' grapefruit trees cultivated under two production systems (**ProdSys**; screenhouse and open-air), two planting systems (**Planting**; in-ground and potted), and two rootstocks (**Rootstock**; Sour orange and US-897).

Source	2015	2016	2017	2018
Model	*0.3643*	*0.0002*	*<0.0001*	*<0.0001*
**ProdSys**	0.4333	<0.0001	<0.0001	<0.0001
**Planting**	0.1454	0.4270	0.0091	0.7946
**ProdSys*Planting**	0.1466	0.3448	0.3538	0.8724
**Rootstock**	0.3419	0.2792	0.2384	0.0311
**ProdSys*Rootstock**	0.6696	0.1925	0.4459	0.0250
**Planting*Rootstock**	0.6851	0.2154	0.0051	<0.0001
**ProdSys*Planting*Rootstock**	0.1346	0.4188	0.0105	<0.0001

Probability (P) values for Soluble Solids Content of 'Ray Ruby' grapefruit trees cultivated under two production systems (**ProdSys**; screenhouse and open-air), two planting systems (**Planting**; in-ground and potted), and two rootstocks (**Rootstock**; Sour orange and US-897).

Source	2016	2017	2018
Model	*<0.0001*	*<0.0001*	*<0.0001*
**ProdSys**	0.0029	<0.0001	<0.0001
**Planting**	<0.0001	<0.0001	<0.0001
**ProdSys*Planting**	0.4494	0.0181	0.0052
**Rootstock**	0.0369	0.0126	0.0106
**ProdSys*Rootstock**	0.0312	0.7119	0.2719
**Planting*Rootstock**	0.3301	0.0051	0.6354
**ProdSys*Planting*Rootstock**	0.5351	.	0.3980

Probability (P) values for Acidity of 'Ray Ruby' grapefruit trees cultivated under two production systems (**ProdSys**; screenhouse and open-air), two planting systems (**Planting**; in-ground and potted), and two rootstocks (**Rootstock**; Sour orange and US-897).

Source	2016	2017	2018
Model	*<0.0001*	*0.0056*	*0.017*
**ProdSys**	<0.0001	0.6612	0.0027
**Planting**	0.9525	0.0258	0.0922
**ProdSys*Planting**	0.0052	0.1834	0.0728
**Rootstock**	0.7660	0.0162	0.2831
**ProdSys*Rootstock**	0.4414	0.9346	0.1360
**Planting*Rootstock**	0.0061	0.1192	0.2576
**ProdSys*Planting*Rootstock**	0.014	.	0.9701

Probability (P) values for Ratio of 'Ray Ruby' grapefruit trees cultivated under two production systems (**ProdSys**; screenhouse and open-air), two planting systems (**Planting**; in-ground and potted), and two rootstocks (**Rootstock**; Sour orange and US-897).

Source	2016	2017	2018
Model	*<0.0001*	*0.0002*	*0.0300*
**ProdSys**	<0.0001	0.0018	0.0930
**Planting**	<0.0001	0.2552	0.0382
**ProdSys*Planting**	0.0217	0.9612	0.0122
**Rootstock**	0.1372	0.0001	0.8673
**ProdSys*Rootstock**	0.1915	0.7901	0.4677
**Planting*Rootstock**	0.0101	0.6372	0.3760
**ProdSys*Planting*Rootstock**	0.0418	.	0.5741

Probability (P) values for Yield of solids of 'Ray Ruby' grapefruit trees cultivated under two production systems (**ProdSys**; screenhouse and open-air), two planting systems (**Planting**; in-ground and potted), and two rootstocks (**Rootstock**; Sour orange and US-897).

Source	2016	2017	2018
Model	*<0.0001*	*<0.0001*	*<0.0001*
**ProdSys**	<0.0001	<0.0001	<0.0001
**Planting**	0.7244	0.0388	0.8629
**ProdSys*Planting**	0.5123	0.6598	0.7546
**Rootstock**	0.9314	0.2778	0.1233
**ProdSys*Rootstock**	0.8421	0.0160	0.1031
**Planting*Rootstock**	0.6570	0.0070	0.0012
**ProdSys*Planting*Rootstock**	0.6293	.	0.0015
